# Condom use at last sex by young men in Ethiopia: the effect of descriptive and injunctive norms

**DOI:** 10.1186/s12978-018-0607-3

**Published:** 2018-10-03

**Authors:** Aparna Jain, Elizabeth Tobey, Hussein Ismail, Annabel Erulkar

**Affiliations:** 1 Population Council, 4301 Connecticut Avenue NW, Suite 280, Washington DC, 20008 USA; 2 Population Council, Heritage Plaza, 4th floor, Bole Medhaneialem Road, Addis Ababa, Ethiopia

**Keywords:** Social norms, Condom use, Theory of normative social behavior, Young men, Ethiopia

## Abstract

**Background:**

Condoms are an important prevention method in the transmission of HIV and sexually transmitted infections as well as unintended pregnancy. Individual-level factors associated with condom use include family support and connection, strong relationships with teachers and other students, discussions about sexuality with friends and peers, higher perceived economic status, and higher levels of education. Little, however, is known about the influence of social norms on condom use among young men in Ethiopia. This study examines the effect of descriptive and injunctive norms on condoms use at last sex using the theory of normative social behavior.

**Methods:**

A cross-sectional survey was implemented with 15-24 year old male youth in five Ethiopian regions in 2016. The analytic sample was limited to sexually active single young men (*n* = 260). Descriptive statistics, bivariate and multivariate logistic regressions were conducted. An interaction term was included in the multivariate model to assess whether injunctive norms moderate the relationship between descriptive norms and condom use.

**Results:**

The descriptive norm of knowing a friend who had ever used condoms significantly increased respondents’ likelihood of using condoms at last sex. The injunctive norm of being worried about what people would think if they learned that the respondent needed condoms significantly decreased their likelihood to use condoms. The injunctive norm did not moderate the relationship between descriptive norms and condom use. Young men who lived closer to a youth friendly service (YFS) site were significantly more likely to have used condoms at last sex compared to those who lived further away from a YFS site.

**Conclusions:**

Social norms play an important role in decision-making to use condoms among single young men in Ethiopia. The interplay between injunctive and descriptive norms is less straightforward and likely varies by individual. Interventions need to focus on shifting community-level norms to be more accepting of sexually active, single young men’s use of condoms and need to be a part of a larger effort to delay sexual debut, decrease sexual violence, and increase gender equity in relationships.

## Plain English summary

Condoms are an important tool to prevent the spread of HIV, sexually transmitted infections, and unwanted pregnancies. Research conducted in Ethiopia and elsewhere has shown various factors that influence young men’s use of condoms. One important but understudied factor is the effect of social norms on condom use. This study assessed how social norms influenced whether young men in Ethiopia used condoms the last time they had sex by applying the theory of social normative behavior. Results showed that social norms do affect condom use: young men who knew of friends who had used condoms were more likely to have used condoms at last sex, and young men who were not worried about what people would think of them if they found out they needed condoms were more likely to have used condoms as well. Interventions should look to change community norms by increasing the acceptance of condom use among young men who are sexually active and not married, and this should be a part of a larger effort to delay the age of first sex, reduce sexual violence, and promote health masculinities and gender equity.

## Background

Eastern and Southern Africa have the highest HIV prevalence rates among young people aged 15–24 in the world with 3.4% of young women and 1.6% of young men living with HIV in 2016 [1]. Though Ethiopia has a relatively low HIV prevalence rate for this region, at 0.4% of young women and 0.5% of young men (UNAIDS 2017) [1], it has the second-highest population in Africa (after Nigeria) at 105 million people in mid-2017 [2]. This large population translates to a high burden of people living with HIV across Ethiopia: in 2016, there were 87,000 young people living with HIV and 8700 new cases among young people across the country [1].

In 2015, the World Health Organization, UNAIDS, and UNFPA issued a position statement encouraging the promotion of condoms to young people, among other populations, as a critical intervention for preventing the spread of HIV, sexually transmitted infections (STIs), and unintended pregnancies [3]. Condom use is also an important indicator for assessing never-married young men’s access to family planning and reproductive health (FP/RH) services. Among Ethiopian men aged 25–49, the median age at first marriage is 23.7 and the median age at first sex is 21.2, 2.5 years before marriage [4]. Furthermore, of the 13.8% of never-married men have who had sexual intercourse in the past 12 months with a person who was neither their wife nor a partner who lived with them, only slightly more than half (53.9%) reported using a condom during last sex with such a partner [4].

Several studies have assessed individual-level factors associated with condom use among young men in Ethiopia. Factors that have shown to support the use of condoms include family support and connection, strong relationships with teachers and other students, discussions about sexuality with friends and peers, higher perceived economic status, and higher levels of education [5–7]. Risk factors associated with non-use of condoms or other risky sexual behaviors include low involvement in religious activities, high levels of alcohol consumption, and poor knowledge of HIV/AIDS [5, 8]. Thus, there is a need to focus on increasing use of condoms among young men, for the prevention of HIV, STIs, and unintended pregnancy. There is also a need for interventions to focus on the contextual factors surrounding pre-marital sex by addressing violence in early sexual encounters, and gender equity.

Evidence on the role of social norms in young men’s decision making to use condoms is mixed. A study of young adults in rural Ethiopia found that social norms influenced intention to use condoms but not reported use of condoms [9]. In four studies in South Africa, Tanzania, Uganda and Swaziland, where norms were measured by different statements, social norms were shown to influence male and female adolescents’ use of condoms [10–13]. However, a study of adolescents in rural Tanzania that examined the role of social norms through statements including the following: “I agree with the opinion of my friends that I should use condoms when having sex” found that norms did not directly predict condom use [14]. These results are inconclusive and do not capture the full range of social norms that can influence behavior.

### Theory of normative social behavior

The theory of normative social behavior (TNSB) [15] is a framework used to explain how social norms influence behavior. TNSB distinguishes between two types of social norms: descriptive norms, which are individuals’ perceptions about the prevalence of a behavior [16], and injunctive norms, which are perceived social pressures to conform [17, 18]. Injunctive norms influence behavior because failure to conform carries the threat of social sanctions [15]. TNSB holds that both descriptive and injunctive norms directly affect behavior, but that the relationship between descriptive norms and behavior is moderated by injunctive norms, among other factors [15]. This theory has been tested for various health behaviors, including contraceptive use [19], alcohol consumption [20], handwashing [21], and physical activity [22].

The purpose of this study is to explore the role of social norms, both descriptive and injunctive, on condoms use at last sex, and to determine whether injunctive norms moderate the relationship between descriptive norms and condom use among young men in Ethiopia.

## Methods

### Data

A cross-sectional household survey of 15–24-year-old males living in rural and peri-urban Ethiopia was conducted in Amhara, Benishangul-Gumuz, Oromia, Southern Nations, Nationalities, and Peoples’ Region (SNNP), and Tigray regions from January to July 2016. The sampling strategy was designed to measure the effect of distance to a youth friendly service (YFS) health center on utilization of a range of health services. Of 247 eligible YFS sites identified in these regions by the Regional Health Bureaus, 5% were randomly selected for inclusion and the number of sites in each region was determined by probability proportional to size. A total of 14 YFS sites were selected from five regions. One non-YFS health center was randomly selected from each region for comparison.

A stratified, two-stage cluster design was employed where enumeration areas (EAs) were the sampling unit for stage one and selected within 5 km of YFS site, within 5 km of a non-YFS site, and within 5-10 km of a YFS site. Households comprised the second stage and approximately 37 households with eligible respondents were randomly selected per EA, and one respondent per household was selected using a Kish grid [23]. A more detailed description of the sampling strategy is available in the study report [24]. The total number of males interviewed was 1244.

The questionnaires included modules on background characteristics; household characteristics; social cohesion and autonomy; puberty, family planning, and sexual activity; and facility visits for condoms, sexually transmitted infections, HIV, and basic health services. The questionnaires were translated into Amharic, Afan Oromo, and Tigrigna.

### Sample population

The sample was limited to 15–24 year old never-married young men who had ever had sexual intercourse. Young men who were married/in-union were excluded from the analysis because they reported low condom use at last sex (1.3%) because they were trying to get pregnant, were in a steady/committed relationship, or because of religious prohibition.

### Measures

The dependent variable is condom use at last sex measured by responses to the question “Did you use a condom the last time you had sexual intercourse?”. Though this measure does not capture consistent condom use, it is advantageous as it minimizes recall bias by only asking about one recent incidence of sex [25]. Respondents who used a condom at last sex were coded as 1 and those who did not use a condom were coded as 0.

Descriptive norms were measured by asking respondents whether they knew of any friend who had ever used a condom. Respondents who knew of a friend who had ever used condoms were coded as 1 and those who did not know of a friend or were unsure were coded as 0.

Injunctive norms were measured by the following attitudinal statement: “I would be worried about what people in my community would say about me if they found out I needed condoms.” This statement was measured on a four-point Likert scale ranging from strongly agree to strongly disagree. The responses were combined to form a dichotomized variable of agree or worry coded as 0 and disagree or not worried coded as 1.

Additional independent variables included in the model were: respondent’s age; education; religion; wealth quintile; ownership of personal savings to assess financial autonomy; living with both parents; chewing khat, drinking alcohol, or smoking cigarettes in the past month; distance living away from a YFS facility to assess physical access and presence of age-appropriate services; age at first sex; and whether the respondent had gone for HIV testing or counseling in the last 6 months.

### Analysis

Descriptive statistics were calculated for respondent characteristics, dependent and independent variables. Bivariate analyses of condom use at last sex were conducted using Pearson’s chi-squared tests and t-tests for significance. Multivariate logistic regression models were used and were adjusted by variables that were statistically significant in the bivariate analysis or were theoretically important. Akaike’s Information Criterion (AIC) was employed to compare relative quality and fit of several models, and the model with the lowest AIC was chosen. The final model was run with and without an interaction term to assess the moderating effect of injunctive norms on descriptive norms. The likelihood-ratio test was used to determine if inclusion of the interaction term improved model fit. Lastly, the Hosmer-Lemeshow test was applied to the final model to assess model fit to the data. All analyses were conducted using Stata v15.

## Results

Table 1 shows characteristics of sexually active single young men aged 15–24 (*n* = 260). Three out of five (60%) respondents were aged 20–24 years and the median age at first sex was 17 years. Most respondents were out of school at the time of the survey (70%), were living with both parents (64%), and did not have their own savings (72%). Approximately half were Orthodox Christian (49%) and the remaining half were Muslim (41%) or Protestant (10%). Over one-third (37%) of respondents lived within 5 km of a YFS site while 38% lived within 5-10 km, and the remaining quarter (25%) lived within 5 km of a non-YFS site. Respondents were of all wealth quintiles, though the greatest proportion was in the lowest wealth quintile (27%).

**Table 1:** Respondent Characteristics (*n* = 260)

	Percent	Number
Age		
15–19	40.8	106
20–24	59.2	154
Age at first sex (median, range)	17.0	(10–23)
School status		
In school	30.4	79
Out of school	69.6	181
Education^a^		
Never attended school	4.6	12
Primary	55.0	143
Secondary/technical/vocational/higher	40.0	104
Living with both mother and father		
No	35.8	93
Yes	64.2	167
Has own savings		
No	71.5	186
Yes	28.5	74
Religion		
Muslim	41.2	107
Orthodox Christian	48.8	127
Protestant	10.0	26
Region		
SNNP	24.2	63
Tigray	21.2	55
Amhara/Benishangul-Gumuz	17.3	45
Oromia	37.3	97
Proximity to health facility/YFS		
< 5 km from YFS	36.5	95
5-10 km from YFS	38.5	100
< 5 km from non-YFS	25.0	65
Wealth quintile		
Lowest	26.5	69
Lower	14.2	37
Middle	19.2	50
Higher	20.4	53
Highest	19.6	51
Chewed khat, drank alcohol, smoked cigarettes in past month		
Did not do any behavior	49.2	128
Did one or more behaviors	50.8	132
Has ever heard of HIV		
No	0.4	1
Yes	99.6	259
Knows HIV can be transmitted via unprotected sex^a^		
No	3.5	9
Yes	96.2	250
Has gone for HIV testing or counseling in last 6 months		
No	89.2	232
Yes	10.8	28
Descriptive norm: Knows a friend who has ever used condoms		
No/don’t know	43.5	113
Yes	56.5	147
Injunctive norm: “I would be worried about what people in my community would say if they found out I needed condoms”		
Disagree	37.3	97
Agree	62.7	163
Ever used of condoms		
No	33.8	88
Yes	66.2	172
Condom use at last sex		
No	44.2	115
Yes	55.8	145

^*a*^Percent and number do not add to 100% or 260 due to missing values

Slightly more than half of the sample had either chewed khat, drank alcohol, and/or smoked cigarettes (51%) in the past month. Most respondents had not gone for HIV testing or counseling in the last 6 months (90%), while virtually all respondents knew of HIV (99.6%) and knew that HIV can be transmitted by unprotected sex (96%). Fifty-seven percent of respondents knew of a friend who had ever used a condom (descriptive norm) and 63% agreed that they would be worried about what people in their community would say if they found out the respondent needed condoms (injunctive norm). Two-thirds (66%) of the respondents had ever used condoms and 56% of the sample used a condom at last sex.

Table 2 presents the results of the bivariate analyses for condom use at last sex. Young men who had attended secondary education or higher were significantly more likely to report condom use at last sex (65%) than those had never attended or only attended primary school (49%). Young men who lived < 5 km from a YFS site were significantly more likely to have used a condom at last sex (65%) compared to those who lived < 5 km from a non-YFS (55%) and those who lived 5-10 km away from a YFS site (47%). Young men living in higher wealth quintiles were more likely than those in lower wealth quintiles to report condom use at last sex, with a range from 67% in the highest wealth quintile to 41% in the lowest.

**Table 2:** Bivariate analysis of condom use at last sex (*n* = 260)

	Condom use at last sex (%)
Age	
15–19	59.4
20–24	53.2
School status	
In school	60.8
Out of school	53.6
Education	
Never attended school/Primary	49.0
Secondary/technical/vocational/higher	65.4*
Living with both mother and father	
No	58.1
Yes	54.5
Has own savings	
No	52.2
Yes	64.9
Religion	
Orthodox	50.4
Muslim	61.7
Protestant	57.7
Region	
Oromia	53.6
Tigray	50.9
Amhara/Benishangul-Gumuz	53.3
SNNP	65.1
Proximity to health facility/YFS	
< 5 km from YFS	65.3*
5-10 km from YFS	47.0
< 5 km from non-YFS	55.4
Wealth quintile	
Lowest	40.6
Lower	54.1
Middle	52.0
Higher	69.8
Highest	66.7**
Chewed khat, drank alcohol, smoked cigarettes in past month	
Did not do any behavior	58.6
Did one or more behaviors	53.0
Has gone for HIV testing or counseling in last 6 months	
No	54.7
Yes	64.3
Descriptive norm: Knows a friend who has ever used condoms	
No/don’t know	40.7
Yes	67.3**
Injunctive norm: “I would be worried about what people in my community would say if they found out I needed condoms”	
Disagree	64.9
Agree	50.3*

* *p*-value ≤0.05; ** *p*-value ≤0.01

In terms of norms, condom use at last sex was significantly associated with both descriptive and injunctive norms. Among those who knew of a friend who had ever used a condom (descriptive norm), 67% used a condom at last sex, compared to 41% of those who did not know of a friend who had used condoms. Condom use at last sex was significantly higher among young men who disagreed with the measure of injunctive norm: those who disagreed (that is, would not worried about what people would say if they found out the respondent needed condoms) were significantly more likely to have used condoms at last sex (65%) than those who agreed (50%).

A t-test was conducted to determine if mean age at first sex differed for those who used condoms at last sex and those who did not. The mean age was 17.2 for both populations, and the difference was not significant (data not shown). Since nearly all respondents reported HIV awareness and knowledge that unprotected sex can transmit HIV, these two measures were excluded from bivariate and multivariate analysis.

Table 3 presents the adjusted odds ratios (AORs) of the effect of descriptive and injunctive norms on condom use at last sex, adjusting for respondent characteristics, distance to a YFS facility, and use of khat, alcohol, or cigarettes. Though theoretically relevant and shown elsewhere as significant predictors to condom use at last sex, we removed five variables from the multivariate model because these measures were not significant in the bivariate analysis, and to ensure a stronger fit of the data: age at first sex, school status, living with mother and father, has own savings, and used HIV testing or counseling in the past 6 months.

**Table 3:** Adjusted odds ratios of condom use at last sex (*n* = 260)

	AOR	95% CI
Age		
15–19	ref	
20–24	0.61	(0.33–1.12)
Education		
Never attended school/Primary	ref	
Secondary/technical/vocational/higher	1.84	(0.99–3.42)
Religion		
Orthodox Christian	ref	
Muslim	2.07	(0.96–4.45)
Protestant	1.63	(0.51–5.23)
Region		
Oromia	ref	
Tigray	1.12	(0.46–2.71)
Amhara/Benshangul-Gumuz	1.07	(0.44–2.63)
SNNP	1.67	(0.72–3.87)
Proximity to health facility/YFS		
< 5 km from YFS	ref	
5-10 km from YFS	0.46*	(0.23–0.92)
< 5 km from non-YFS	0.47	(0.22–1.05)
Wealth quintile		
Lowest	ref	
Lower	2.31	(0.87–6.14)
Middle	1.64	(0.70–3.85)
Higher	3.10*	(1.24–7.73)
Highest	1.99	(0.79–4.99)
Chewed khat, drank alcohol, smoked cigarettes in past month		
Did not do any behavior	ref	
Did one or more behaviors	0.88	(0.45–1.70)
Descriptive Norm: Knows a friend who has ever used condoms		
Yes	4.74**	(2.26–9.95)
Injunctive norm: “I would be worried about what people in my community would say if they found out I needed condoms”		
Disagree	3.39**	(1.38–8.35)
Descriptive norm × Injunctive norm		
Descriptive Norm: Yes × Injunctive Norm: Disagree	0.37	(0.12–1.19)

* *p*-value ≤0.05; ** *p*-value ≤0.01

The likelihood-ratio test was used to test the difference between the model with the interaction term of injunctive and descriptive norms and the model without the interaction. The test was borderline significant with a *p*-value of 0.0952. The final model presented in Table 3 includes the interaction term.

Among respondents who agreed with the injunctive norm statement (would be worried about what people in their community would say if they found out they needed condoms), those who knew of a friend who had ever used condoms were 4.7 times (95% CI: 2.26–9.95) more likely to use condoms at last sex than those who did not have a friend who had used condoms. Furthermore, among respondents who did not know of a friend who had ever used a condom, those who disagreed with the injunctive norm statement (or would not be worried) were 3.4 times (95% CI: 1.38–8.35) more likely to have used a condom at last sex compared to those who agreed with the statement. The interaction term of descriptive norm × injunctive norm was not statistically significant, and no synergistic effect of condom use at last sex was observed.

Respondents who lived 5-10 km away from a YFS facility were significantly less likely to have used a condom at last sex compared to those who lived within 5 km of a YFS site (AOR = 0.46, 95% CI: 0.23–0.92). There was no significant difference in the odds of condom use at last sex for those who lived within 5 km from a non-YFS site compared to those who lived within 5 km from a YFS site. No significant difference was observed among those who lived 5-10 km from YFS to those who lived < 5 km from non-YFS in condom use at last sex (data not shown). Those who lived in the higher wealth quintile were significantly more likely to have used a condom at last sex than those in the lowest quintile (AOR = 3.10, 95% CI: 1.24–7.73).

The Hosmer-Lemeshow goodness-of-fit test demonstrated that the model fits the data reasonably well (*p* = 0.601).

## Discussion

The purpose of this study was to assess the role of social norms in condom use at last sex among sexually active single young men in Ethiopia. Using the TNSB, the study looked at how perceptions of friends’ use of condoms (descriptive norm) and worry of what community members would say if they learned that the respondent needed condoms (injunctive norm) influenced condom use at last sex. This study also explored whether the injunctive norm moderated the relationship between the descriptive norm and condom use. The results show that, in the context of never-married, sexually active young men in Ethiopia, while descriptive and injunctive norms individually influence condom use at last sex, injunctive norms do not moderate the relationship between descriptive norms and condom use.

A study of contraceptive use using the TNSB framework in India also found that injunctive norms do not have a strong moderating effect on the relationship between descriptive norms and behavior [19]. Condom use, like contraceptive use, is a semi-private behavior in that it generally occurs between partners with limited public knowledge of the behavior. The lack of moderation may have occurred because private behaviors are not observed by others and are thus not subjected to the same levels of public scrutiny or stigma as public behaviors. In addition, the lack of moderation on condom use suggests that descriptive and injunctive norms may function differently for individuals and their interaction is more complex.

The study results are important for the Ethiopian government to reach its ambitious goals related to HIV knowledge, condom use and HIV testing and counseling by 2020 [26]. Different programmatic strategies with young men need to be tested and evaluated. For instance, the Ethiopian government is exploring the inclusion of FP/RH and HIV education in school-based programming [26]. In our sample, the vast majority (95%) had attended at least primary school, though only 40% went on to secondary school. Using primary schools as a space where age-appropriate information can be shared about FP/RH and HIV at earlier ages may provide young men with the knowledge and tools that they need to make better and safer decisions around condom use in the future.

The study results also showed that respondents who are worried and concerned about how they would be perceived if others in their community learned that they needed condoms suggest that even in a place like Ethiopia, where condoms are relatively ubiquitous, fear of how one who uses condoms is perceived can weigh heavily on adopting protective behaviors. As has been shown with programs to delay girls’ marriage [27, 28], the Ethiopian government may consider holding community conversations to engage young men and their parents to begin addressing social norms that restrict adolescents from using condoms, especially in rural areas where traditional notions forbidding pre-marital sex exist.

The study showed that unmarried young men who know of a friend who has used condoms are more likely to use condoms themselves suggests that communication and sharing of information within social and peer networks is important in changing behaviors. Condom use is by and large a private behavior that is not explicitly known or seen by others. Knowledge of a friend’s use of condoms would likely occur in discussions and so if young men brag to their friends about sex [29, 30], then perhaps the narrative around sexual discourse has changed to also include condom use. A study in Ethiopia showed that discussions about sexuality with friends had a positive association with condom use, though “discussions of sexuality” was not well defined [6]. Condom use may be also considered a sign of autonomy or increased status, where young men can obtain condoms without shame. The government may consider creating young mens’ groups to engage unmarried young men in a range of health and relationship issues, including the importance of condoms use, and to provide referrals to facilities for HIV counseling and testing.

The study also showed that respondents who lived closer to a YFS facility were significantly more likely to use condoms at last sex compared to those who lived further away from a YFS facility, and marginally more likely than those who lived close to a non-YFS facility. This finding suggests that proximity to a facility, especially one that has received programmatic interventions to increase its youth friendliness, is important to young men’s use of condoms at last sex. Ethiopia is scaling YFS sites across the country, and this may additionally contribute to increased condom use among young men. The results of this study suggest that increasing YFS, however, is not sufficient to increase condom use - social norms also must be addressed.

Interventions aimed at increasing condom use and addressing social norms should also focus on greater contextual factors in Ethiopia. Because early sexual encounters in Ethiopia are often in the context of force or coercion [6, 31, 32], interventions should consider the role of gender-based violence and inequitable gender norms in condom use. For young women and girls in Ethiopia, the formation of girls’ groups and community conversations about child marriage were shown to be effective not only in raising the age at marriage [27, 28], but also in increasing FP/RH knowledge and voluntary contraceptive use [27]. Adaptations of these interventions may include forming young men’s groups and convening community conversations to engage young men, their parents, and community leaders to begin shifting social norms that inhibit condom use and address gender norms, raise the age of sexual debut, and decrease gender-based violence among young people in Ethiopia.

Further research is necessary especially on positive deviants, that is the young men who did use condoms at last sex, to understand the pathways that lead to them to this decision including where they first heard about condoms, how they learned to use them or where to get them, negotiating with their partner, among many other issues.

Examining the factors associated with condom use at last sex among young males is paramount, as there is evidence to suggest that decision-making on condom use rests predominantly with males [6]. Efforts to examine and increase condom use must therefore include and target boys and young men, as the present study has, and empower them to access and use condoms. However, social change is also critical to enable inclusion of girls and young women in the discourse around condoms, so that condom decision-making becomes more equitable between partners.

### Study limitation

A limitation of this study is the lack of temporality. Because this is a cross-sectional survey, it is not certain that knowledge of friend’s use of condoms influenced condom use at last sex or whether use of condoms at last sex influenced the respondent to discuss condoms with their friends. Either way, communication around condom use with friends and peers appears to be important.

## Conclusions

This study examined condom use at last sex among single young men in rural Ethiopia through the theory of normative social behavior to assess the relationship of descriptive and injunctive norms on behavior. More than half of single young men used condoms at last sex. Those who knew of a friend who had used condoms (descriptive norm), and who were not worried about what members of their community would say if they found out they needed condoms (injunctive norms) were more likely to use condoms at last sex, though there was no moderating effect of injunctive norms. Young men who lived close to a YFS site were also more likely to use condoms at last sex. The results of this study suggest that social change is needed to improve access to and use of condoms at last sex in Ethiopia.

## Abbreviations

AIC: Akaike’s information criterion
AIDS: Acquired immune deficiency syndrome
AOR: Adjusted odds ratio
CI: Confidence interval
FP/RH: Family planning and reproductive health
HIV: Human immunodeficiency virus
SNNP: Southern Nations, Nationalities, and Peoples’ Region
STI: Sexually transmitted infection
TNSB: Theory of normative social behavior
UNAIDS: Joint United Nations Programme on HIV and AIDS
UNFPA: United Nations Population Fund
USAID: United States Agency for International Development
WHO: World Health Organization
YFS: Youth friendly services
